# Sagittal synostosis scaphocephaly cranial reconstruction with spiral cut cranioplasty

**DOI:** 10.3171/2021.1.FOCVID20104

**Published:** 2021-04-01

**Authors:** Catherine Y. Wang, Alisha R. Bonaroti, Brandon A. Miller, James Liau

**Affiliations:** Departments of 1 Neurosurgery and; 2 Plastic Surgery, University of Kentucky, Lexington, Kentucky

**Keywords:** sagittal craniosynostosis, open cranial vault reconstruction, scaphocephaly

## Abstract

Sagittal craniosynostosis, the most common form of craniosynostosis, affects 1 per 1000 live births. The main surgical treatments include endoscopic suturectomy and open cranial vault remodeling. This video describes an open reconstruction method, including strip resection of the sagittal suture, biparietal craniotomies with spiral cut cranioplasty, and barrel staves of the posterior occiput. Ideally used between 4 and 15 months of age, this approach takes advantage of the flexibility of the cranial bones to expand, allowing for immediate and long-term increases of the parietal width and correction of cosmetic deformity, without necessitating the use of cranial molding devices postoperatively.

The video can be found here: https://vimeo.com/516699203

## Transcript

This is a brief summary of sagittal synostectomy scaphocephaly cranial reconstruction using a spinal cut technique. The neurosurgeons are Dr. Brandon Miller and Dr. Wang (resident). The pediatric plastic surgeons are Dr. James Liau and Dr. Bonaroti (resident).

**0:33 Indications**. We find the best time to do this procedure is between 4 months and 15 months of age, as the spiral technique is predicated upon the flexibility of the cranial bones to expand and not fracture. Before this age, endoscopic approach has good results. After this age, a staged approach with parietal expansion and then frontal bossing correction seems to yield the best results in our hands.

**0:56 Positioning**. The positioning is a modified sphinx/prone position, making sure all pressure points are padded, especially the eyes. Note the rest on foam stuck on the beanbag used as extra protection for the eyes.

**1:10 Initial Incision and Dissection**. The zigzag coronal approach maximizes the camouflage of the scar after the hair growth returns. Using electrocautery, we incised through the skin all the way through down to the pericranium. Use of electrocautery to begin the incision maximizes hemostasis without any noticeable difference in scar formation postoperatively. We then began subperiosteal dissection using a combination of periosteal elevators and blunt dissection. Hemostasis is controlled along the way using bone wax along with electrocautery.

**1:39 Description of Extent of Sagittal Synostosis Resection**. The sagittal synostosis resection is approximately 1.5–2 cm wide, depending on the size of the child.^1,2^ The spinal cut technique focuses on the parietal narrowing of the scaphocephaly. We do not do any frontal bossing reconstruction, as this normalizes with growth after synostectomy.^3^ This limits the amount of overall blood loss, as well as allows better visualization intracranially during the craniotomy. Note that the craniotomy stays within the lambdoid, coronal, and squamosal suture lines.

**2:06 Sagittal Synostectomy**. The craniotomy consists of supradural dissection of the anterior fontanelle. We also perform controlled burr holes of the posterior sagittal synostosis. Kerrison rongeurs then allow controlled osteotomy across the synostectomy site. During the sagittal synostectomy, previous dissection has freed up the dura and the sagittal sinus, thus allowing the footplate of the craniotome to remain superficial to the structures and avoid injury.

**2:36 Parietal Craniotomies**. During the parietal craniotomy, the co-surgeon is directly visualizing the footplate while simultaneously dissecting away the dura. This ensures complete intracranial control and direct visualization of the dura prior to the craniotomy, thus preventing dural injury. This is especially pertinent when cutting above the squamosal suture due to inherent dural adhesion to suture lines.

**3:02 Barrel Staves of Posterior Occiput**. Barrel staves of the posterior occiput allow optimal flaring of the posterior cranium to assist in the parietal widening during growth.

**3:12 Spiral Cuts**. The spiral cuts in the parietal bone need to be thick enough so that there is no risk of fracture; however, must be thin enough to ensure adequate flexibility to accordion out. We find that three spirals work the best. Note how much the bone is able to expand, yet there is no risk of bone fracture. We have also found that the flexibility of bone begins to decrease after 1 year of age.

**3:40 Cranioplasty**. Using resorbable polymer mesh and securing it with a rivet system is an efficient method of securing the parietal bone back in situ. We also secure the superior aspect to make sure there is no overlap of the superior cranial bone and to prevent excessive cranial contour abnormality as the child heals. Note that the brain has already begun to push out on the spiral cuts intraoperatively resulting in expansion.

**4:05 Closure**. Closure is without tension and done in a two-layer fashion with subcutaneous tissue and galea reapproximation with 4-0 resorbable suture, and skin closure with a 5-0 plain gut running simple closure.

**4:19 Postoperative Result**. You can tell immediately intraoperatively the amount of parietal width increase, and continued widening in the before and after photos. As the child continues to grow, the parietal width continues to widen into a normal cranial index.^4,5^

## Author Contributions

Primary surgeon: Liau. Assistant surgeon: Bonaroti, Miller. Editing and drafting the video and abstract: Wang, Liau. Critically revising the work: Wang, Bonaroti, Miller, Liau. Reviewed submitted version of the work: all authors. Approved the final version of the work on behalf of all authors: Wang. Supervision: Wang, Miller, Liau.
